# Cellulose citrate: a convenient and reusable bio-adsorbent for effective removal of methylene blue dye from artificially contaminated water†

**DOI:** 10.1039/d1ra05464c

**Published:** 2021-10-22

**Authors:** Fabrizio Olivito, Vincenzo Algieri, Antonio Jiritano, Matteo Antonio Tallarida, Antonio Tursi, Paola Costanzo, Loredana Maiuolo, Antonio De Nino

**Affiliations:** Laboratory of Organic Synthesis and Chemical Preparations (LabOrSy), Department of Chemistry and Chemical Technologies, University of Calabria Rende CS Italy fabrizio.olivito@unical.it vincenzo.algieri@unical.it denino@unical.it; Laboratory of Physical Chemistry, Materials and Processes for Industry, Environment and Cultural Heritage (CF-INABEC), Department of Chemistry and Chemical Technologies, University of Calabria Rende CS Italy

## Abstract

In the present work, we proved the efficacy of cellulose citrate to remove methylene blue (MB) from artificially contaminated water. MB is a widely used dye, but because of its chemical aromatic structure, it is significantly stable with quite slow biodegradation, causing consequent serious health problems for people and significant environmental pollution. Cellulose citrate, the bio-adsorbent proposed and studied by us to remediate water polluted by MB, is produced by a green, cheap and fast procedure that makes use of two abundant natural products, cellulose and citric acid. The average of two citrate groups for each glucose unit of cellulose chains allows this material to have many carboxylic groups available for interaction with the cationic dye. The characterization was carried out through FT-IR, SEM, specific surface area, pore structure parameters and zeta potential. The negative value of the zeta potential at neutral pH is consistent with the affinity of this material for the adsorption of cationic compounds like MB. The activity of the adsorbent at different times, temperatures, pH and concentrations was investigated. The process followed monolayer adsorption typical of the Langmuir model, with a maximum adsorption capacity of 96.2 mg g^−1^, while for the kinetic studies the process followed a pseudo-second order model. The highest levels of adsorption were reported using solutions of dye with concentrations under 100 mg L^−1^. The adsorbent can be regenerated several times without a significant loss in the adsorption capacity, and it is not strongly affected by temperature and pH, giving rise to a simple and eco-sustainable procedure for water remediation. Therefore, we conclude that cellulose citrate can be considered as a promising bio-adsorbent for the removal of MB and other cationic pollutants from the environment.

## Abstract 2

In the present work, we proved the efficacy of cellulose citrate to remove methylene blue (MB) from artificially contaminated water.

## Graphical abstract

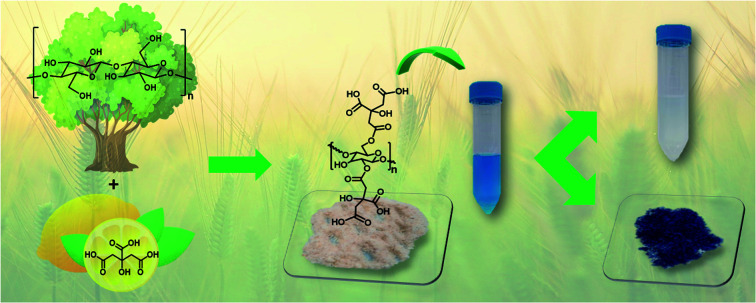

## 1 Introduction

In recent years there has been increasing and widespread concern regarding environmental pollution and, therefore, the effort of the scientific community to solve these problems has been remarkable. The number of research papers about air, water, and land treatment strategies for pollutant removal has grown enormously.^1–6^ Dyes are very common in different industries, like cosmetics, food processing, paper and pulp, pharmaceutics, agriculture, and textiles.^7,8^ Their use falls within a well-established strategy to prepare functional materials with added-value properties by combination with other materials at the nanoscale.^9^ Particularly, organic dyes represent the major pollutants of textile wastewater.^10^ In fact, natural colorants were substituted throughout the years by more reactive synthetic ones because they produce an easy, fast and long-lasting dying of the fabric fibers, sometimes due also to the formation of covalent bonds.^11^ However, the general aromatic structure of these compounds makes them exceptionally stable with a quite slow biodegradation.^12,13^ Methylene blue (MB) is an organic chloride salt having a thiazine structure condensed with two aromatic rings. Methylene blue is widely used as a dye in paper, silk, cosmetics, chemical laboratory procedures and in human and veterinary medical procedures.^14–18^ Anyway, MB exhibits a severe level of toxicity for humans, principally *via* oral, inhalation, injection, or intravenous exposure.^19^ In particular, it can induce damage to red blood cells and decrease the ability of the blood to carry oxygen (methemoglobinemia). Moreover, prolonged exposure to MB can cause, among other things, headaches, difficulty breathing, abnormal heartbeat, and allergic reactions. On the other hand, also the effects on the environment are not negligible, because its elevated quantity in wastewater induces a minor light absorption to detriment of the photosynthetic process of aquatic plants and significant toxicity for the aquatic organisms.^20,21^ In spite of everything, methylene blue is surely one of the most used dye in textile industries and, for this reason, the developing of new strategies for the efficient removal from water effluents is still ongoing and of great interest.^22–27^ The common methods for the removal of organic dyes include: chemical oxidation or reduction,^28^ precipitation,^29^ ion exchange,^30^ electrolysis,^31^ photo-catalytic processes,^32,33^ membrane filtration,^34^ ozonation,^35^ degradation,^36^ bio-sorption^37^ and adsorption.^38,39^ In particular, adsorption is one of the most used techniques because it relies on a simple mechanism, does not produce handling problems, and the process can be reversible.^40,41^ Among adsorbents, activated carbon was employed over the years because of its efficacy for the removal of water and air pollutants, but it possesses many limitations as the production costs and the regeneration.^42,43^ The conversion of waste into bio-adsorbent has a high economic value other to generate a very low impact on the environment with an easy biodegradability.^44,45^ Inthapanya and co-workers investigated the removal of anionic acid green 25 using calcined oyster shells with a reported maximum adsorption capacity of 34.1 mg g^−1^.^46^ In another work Lin *et al.* described a study on visible-light-driven photocatalysis for the quantitative degradation of malachite green using a system with silver phosphate modified by MWCNTs and Cr-doped SrTiO_3_.^47^ Pure cellulose and/or cellulose-derived materials were deeply employed in the manufacturing of bio-adsorbent for pollutants removal, due to many advantages like the wide natural availability, chemical versatility, biodegradability, and precious chemical–physical properties.^48–50^ The activity of microcrystalline cellulose for MB adsorption was thoroughly investigated, but although it was proved to be efficient, in all the studies conducted to date, it is quite far from a quantitative removal.^51–53^ On the other hand, in literature was reported that the functionalization of cellulose-based materials and biomass waste with carboxylic groups, enhances the adsorption capacity, even if performed through the use of hazardous materials like chloroacetic acid and methacrylic acid.^54–56^ In this work, we have developed a green, cheap and fast method for the effective removal of MB to remediate polluted water by cellulose citrate (CC) that was obtained through a simple and eco-sustainable reaction between microcrystalline cellulose and citric acid. The synthesis of CC was conducted in open air and makes use of harmless, cheap, and sustainable source reagents.^57^ The application of synthesized cellulose citrate in the removal of MB was almost complete in neutral conditions at an initial dye concentration of 10 mg L^−1^ and the adsorption capacity was retained at higher concentrations with a removal of 58% using a 150 mg L^−1^ of MB solution, with 25 mg of the adsorbent. The mechanism of adsorption, equilibrium, dependency of time, concentrations and pH together with isotherm and kinetic studies were thoroughly investigated during this work. The characterization was carried out through FT-IR, SEM, specific surface area, pore structure parameters and zeta potential.

## 2 Experimental

### 2.1 Reagents

Water, acetonitrile and methanol, all for HPLC analysis in gradient grade ≥99.9%, were purchased from Sigma-Aldrich and degassed before use. Formic acid was purchased from Sigma-Aldrich in gradient grade ≥99.9%. Methylene blue was purchased from Alfa Aesar, in gradient grade ≥99.9%. Microcrystalline cellulose was purchased from Sigma-Aldrich with purity level of 100.

### 2.2 Preparation of cellulose citrate

Cellulose citrate was obtained by a procedure described in our previous work.^57^ Microcrystalline cellulose (10 g) was mixed with citric acid (10 g) (monohydrate or anhydrous) in equivalent weight ratio, in an open air Pyrex flask, and the system heated to 150–155 °C. After 1 hour the mixture was cooled down and washed several times with acetone and then with water to remove the bio-oil adsorbed onto the solid. The collected solid in the form of cellulose citrate was dried in an oven at constant temperature of 70 °C for overnight time.

### 2.3 Average degree of substitution of cellulose citrate

Cellulose-citrate was dispersed in distilled water at 2 mg mL^−1^ at room temperature for 30 min using a sonicator. Subsequently, the pH was adjusted to 10 with a solution 0.1 M of NaOH. The resulting solution was titrated with a solution 0.1 M of HCl, and the change in voltage was monitored using a conductivity meter. Average degree of substitution values was calculated according to ATSM D1439.1 This method consists in dispersing cellulose-citrate in a 0.1 M NaOH solution and titrating with a 0.1 M HCl solution. Degrees of substitution were calculated using the following equation: *G* = 0.162*A*/(1 − *XA*) where *A* = (*BC* − *DE*)/*F* with *B* = mL of NAOH solution added, *C* = *E* = 0.1 (molarities of HCl and NaOH), *F* = amount of cellulose-citrate used in g, 162 = the molar mass of the AGU unit, and *X* = increase in molecular mass for cellulose-citrate (176 for citrate modified cellulose). Values obtained were divided by two due to the fact that each citrate unit introduces two free carboxylic acids. After three hours esterified cellulose starts to decompose and the degree of substitution becomes lower. A time-dependent conductometric titration was carried out.^57^

### 2.4 FT-IR characterization

FT-IR spectra of microcrystalline cellulose (MCC), cellulose citrate (CC), methylene blue (MB) and cellulose citrate after adsorption of methylene blue (MB-CC), were acquired by the Shimadzu IRAffinity-1S spectrometer (Shimadzu Corporation) in the spectral region of 375 and 4000 cm^−1^ with a resolution of 1 cm^−1^, setting 50 scans for a single analysis and using KBr pellets technique. The KBr pellets were obtained by mixing the sample with KBr powder (ratio 1 : 100) and pressing with a hydraulic press, at the pressure of 10 tons for 5 minutes. The resulting pellets were placed in the appropriate compartment of the instrument and exposed to the FT-IR light beam for analysis. Morphological studies of MCC, CC and MB-CC, were carried out using a LEO 420 scanning electron microscope (SEM, Zeiss), operating with vacuum conditions of 8 × 10^−6^ torr at an accelerating voltage of 15 kV. Samples were gold metallized by an Auto Sputter Coater (Agar). Images were taken with 100 and 3000 SEM micrograph magnifications.

### 2.5 Specific surface area and pore structure parameters

Specific surface areas and pore structure parameters of MCC and cellulose citrate were determined utilizing adsorption/desorption N_2_ isotherms at 77 K (Brunauer–Emmett–Teller technique) by means of an ASAP™ 2020 (Micrometrics, Norcross (Atlanta), GA, USA). Prior to the analysis, the dried samples were degassed at 90 °C for 12 h and, then, submitted to N_2_ adsorption at the temperature of liquid nitrogen.^58^ The total pore volume was estimated from the amount of N_2_ gas that was adsorbed at 0.98 of the relative pressure. The pore size distribution was obtained from adsorption/desorption N_2_ isotherms by Barrett–Joyner–Halenda (BJH) method.^59^

### 2.6 Zeta potential measurements

The surface charge of cellulose citrate was analyzed using a zeta potential analyzer (Zetasizer Nano ZSP (ZEN 5600)).

### 2.7 Methylene blue adsorption by cellulose citrate

Samples were analysed using HPLC Agilent series 1100 equipped with an isocratic pump (Agilent technologies 1200 series) and UV-vis detector. In this study we used HPLC equipped with UV-vis detector instead of UV alone, to perform a quantitative test, without the risk of taking into account any decomposition pathway of methylene blue occurring during the batch experiments or other contaminants which could overestimate the data. The reversed phase analytical column used was a C18 Jupiter, with dimensions of 300 A, 250 × 4.60 mm, and 10 micron for particle size (Phenomenex, USA). The wavelength of the reference was 360 nm with a peak width of 100, while the wavelength for the compound of interest for this study was set to 663 nm with a peak width of 16. The chosen mobile phase was 0.01% of formic acid in water (49.99%) and acetonitrile (50%), in isocratic elution mode at a flow rate of 2.5 mL min^−1^. The sample injection volume was 20 μL. The column was washed with methanol after 5 injections for 30 minutes. The adsorption measurements were conducted in Pyrex one neck round bottom flasks of 50 mL, using an Heidolph plate for stirring and heating when it was requested (MR 3001 K). Under heating the flasks were equipped with a condenser. The temperature of the mixture was checked by a temperature probe. The stirring was maintained for all the experiments at 200 rpm. We chose this methodology because allowed an efficient contact between the micro-particles of the adsorbent and methylene blue solution together to a constant temperature, with the achievement of reproducible values. The analysis was conducted in triplicate with comparable values. In addition, at higher temperature respect to room temperature, we used the condenser to avoid any loss of the solvent and as a consequence any change in concentration of the pollutant with the obtaining of wrong measurements. This system has also the advantage of operating under atmospheric pressure. The batch adsorption process was carried out using cellulose citrate as the adsorbent for the removal of methylene blue from water. The adsorbent amount was 25 mg and was added to 25 mL of MB solution 30 mg L^−1^ prepared in distilled water at 20 °C. Aliquots of the solution were withdrawn at different time intervals (0.5–150 min). The concentration of the unabsorbed dye was determined at 663 nm with HPLC coupled to UV-vis detector using a calibration curve of MB made with five points of 1, 5, 10, 20 and 30 mg L^−1^. Control test was carried out before each analysis. Different parameters were studied respect to MB adsorption, as a function of initial MB concentration (10–150 mg L^−1^), pH (3–10), temperature (20–70 °C), using 25 mg of cellulose citrate in 25 mL of MB solution. The pH of neutral solution was 6.2, while the pH of the other solutions was adjusted with HCl 0.1 M and NaOH 0.1 M. The measurements were carried out using a pH meter (HI221, Hanna Instruments), calibrated with three different buffer solutions (pH = 4.01, 7.01 and 10.01). The amount of dye adsorbed on cellulose citrate was calculated in terms of adsorption capacity (*q*) and percent removal (*P*_r_), using the following eqn (1) and (2):^60^1
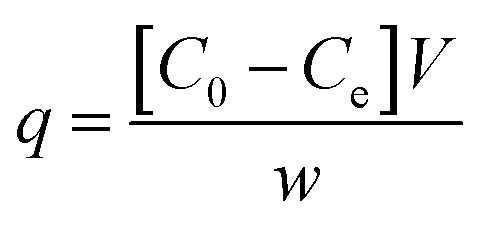
2
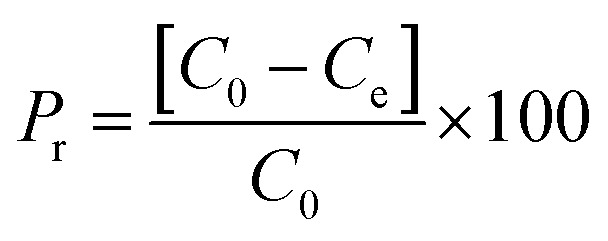
where *q* is the adsorption capacity referred to the amount of the dye adsorbed onto unit dry mass (mg g^−1^), *C*_0_ is the initial concentration of MB in the solution (mg L^−1^), *C*_e_ is the MB equilibrium concentration in the solution at time *t* (mg L^−1^), *V* is the volume of the MB solution (L) and *w* is the weight of cellulose citrate (g). The adsorbent was regenerated and reused using 0.1 M HCl.

## 3 Results and discussion

In our recent paper we defined the optimal reaction conditions to obtain the best average degree of substitution of cellulose citrate. One equivalent of citric acid allows to obtain a value of 0.46 for the glucose unit after a reaction time of 30 minutes. The degree of substitution of cellulose citrate remains the same with higher equivalent of acid.^57^

The adsorption properties of the cellulose citrate were tested on water artificially contaminated by different concentrations of MB. In particular, as already mentioned in the experimental section, initial tests were conducted on a solution of 25 mg of CC added to 25 mL of MB solution (30 mg L^−1^). The significant achieved results were extended to solutions at several MB concentrations (10–150 mg L^−1^). In addition, dependency of time, temperature and pH together with isotherm and kinetic studies were deeply investigated to verify the effectiveness and the excellent applicability of our method to removal MB dye from polluted water.

### 3.1 Materials characterization

#### 3.1.1 Specific surface area and pore structure parameters

The values for the specific surface area (BET SSA), total pore volume and particles dimension of the MCC and cellulose-citrate are given in Table 1.

**Table 1:** Parameters relative to MCC and cellulose citrate

Parameter	MCC	Cellulose citrate
BET SSA	1.311 ± 0.061 m^2^ g^−1^	1.854 ± 0.079 m^2^ g^−1^
Total pore volume	0.0154 ± 0.0009 mL g^−1^	0.0202 ± 0.0008 mL g^−1^
Particles dimension^a^	15–225^a^ μm	5–130^a^ μm

a Estimated from the SEM micrographs.

The specific surface area (BET SSA) results show a significant increase of this parameter (more than 30%) in cellulose citrate compared to MCC, following the hydrolysis/functionalization process. As well as, the total pore volume results registered an increased porosity in cellulose citrate of about 25% *vs.* MCC. Furthermore, the BJH method depicted that the majority of pores were distributed under 50 nm in both samples with an average value of 21 nm and 19 nm for MCC and cellulose citrate, respectively, indicating that materials are mesoporous in nature (mesopores are comprised between 2 and 50 nm).^61^

#### 3.1.2 FT-IR characterization

**Fig. 1:**
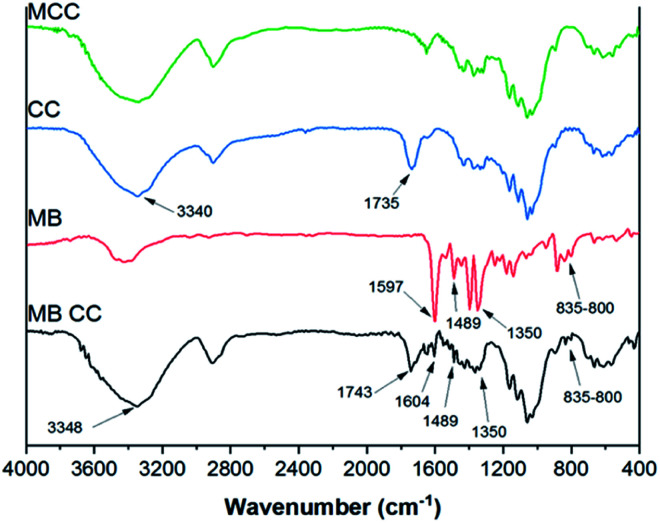
FT-IR spectra of microcrystalline cellulose (MCC, green), cellulose citrate (CC, blue), methylene blue (MB, red), methylene blue adsorbed on cellulose citrate (MB-CC, black).

FT-IR spectra of microcrystalline cellulose (MCC), cellulose citrate (CC), methylene blue (MB), and methylene blue adsorbed on cellulose citrate (MB-CC) are compared in Fig. 1. In the FT-IR spectrum of CC, more in detail, the signal at 3340 cm^−1^ corresponds to O–H stretching and the signal at 1735 cm^−1^ is due to ester carbonyl stretching vibration. Regarding MB spectrum, the signals in the range 1350–1597 cm^−1^ were assigned to the C–C stretching of the aromatic moieties of methylene blue,^62^ whereas the signal due to the methyl groups^63^ is scarcely visible. Finally, the peaks between 800 and 835 cm^−1^ denoted the C–N bending signals.^64^

**Fig. 2:**
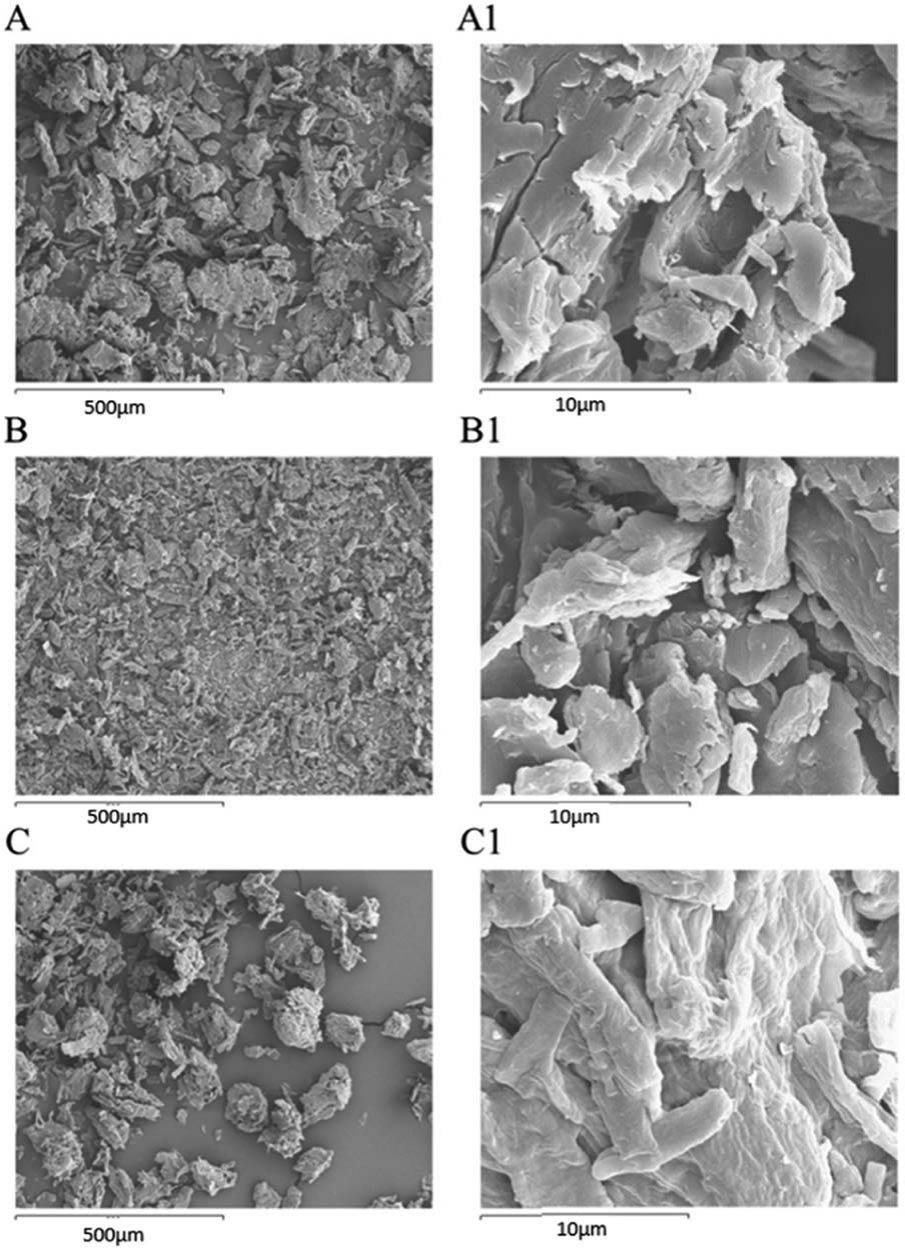
SEM images: (A) MCC [15 kV × 100], (A1) MCC [15 kV × 3000], (B) CC [15 kV × 100], (B1) CC [15 kV × 3000], (C) MB CC [15 kV × 100], (C1) MB CC [15 kV × 3000].

After the MB adsorption on cellulose citrate (MB-CC spectrum), the characteristic aromatic peaks of the dye appeared in the range 1350–1597 cm^−1^, together with the C–N bending band between 800 and 835 cm^−1^.^65^ In particular, in the MB-CC spectrum, the MB aromatic peak at 1597 cm^−1^ is shifted at 1604 cm^−1^ and the stretching relative to the carbonyl group at 1735 cm^−1^ is shifted at 1743 cm^−1^, respectively. In addition, the stretching band of OH is shifted at 3348 cm^−1^, due to the coordination of methylene blue with hydroxyl groups of cellulose citrate.^66^ These blue shifts can be due to the insertion of MB within the polymeric matrix of CC, since it produces a decrease of inter-chain interactions. Simple statistical considerations may be helpful in this sense: the random insertion of MB into the polymeric matrix of CC would decrease the probability of finding two polymeric segments in close proximity (interacting). In other words, the progressive insertion of MB has the effect of substituting CC–CC interactions with the weaker CC-MB ones: hence, the overall degree of inter-chain interactions is progressively reduced. Such an effect is similar to what found in organic polymers.^67^ The morphological study of MCC, CC, and MB-CC, was investigated by SEM analysis (Fig. 2). The morphology of microcrystalline cellulose MCC (Fig. 2(A) and (A1)), revealed the typical cellulose structure made of cylindrical fibrils forming packed and unpacked agglomerates. The SEM images of cellulose citrate CC (Fig. 2(B) and (B1)), show the evident fragmentation of cellulose chains in smaller fibrils. The adsorption of methylene blue on cellulose citrate (MB-CC) was noticeable from the SEM images (Fig. 2(C) and (C1)). In particular, the cellulosic fibrils coated with methylene blue tend to aggregate, probably due to electrostatic intermolecular attractions (Fig. 2(C)). In addition, the morphology of MB-CC reveals structural compactness, due to the pores that got filled after the dye adsorption (Fig. 2(C1)).

### 3.2 Adsorption kinetics

**Fig. 3:**
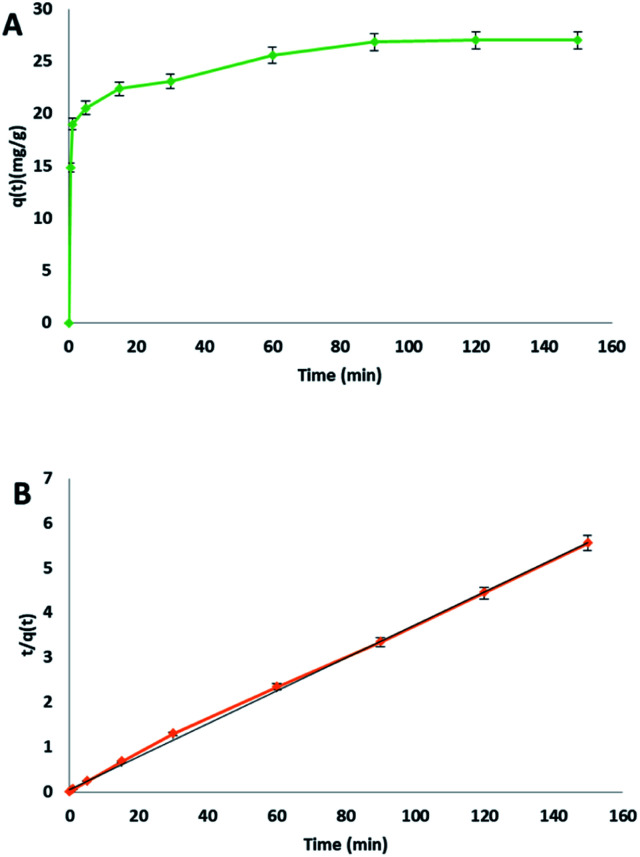
(A) MB adsorption on cellulose citrate with respect to time, (B) pseudo-second order kinetic plot.

The relation between contact time and the adsorption of methylene blue on cellulose citrate was studied employing 25 mg of adsorbent in 25 mL of MB solution with a concentration of 30 mg L^−1^, at 20 °C (Fig. 3(A)).

The MB removal was very fast in 1 minute with an adsorption capacity of 18.99 mg g^−1^, corresponding to 63% removal. This rapid initial adsorption was due to the total availability of the reactive sites on the surface of the adsorbent, in the form of citrate carboxylic groups. From 1 minute to 90 minutes, the removal was slower due to the saturation of the reactive groups on the surface even if the MB removal reaches 90% with an adsorption capacity of 26.86 mg g^−1^. After 90 minutes, the further adsorption followed a plateau because of the complete saturation of the adsorbent. The collected data was fitted using pseudo-first order (see ESI file†), pseudo-second order (Fig. 3(B)) and Elovich model (see ESI file†) for the kinetic plots. The obtained parameters from the different kinetics plots are listed in Table 2.

**Table 2:** Parameters of different kinetic models for methylene blue adsorption on cellulose citrate (MB-CC)

Kinetic model	Parameter	Value
Pseudo-first-order	*k*_1_	1.4 × 10^−2^ min^−1^
	*q*_e_	2.57 mg g^−1^
	*R* ^2^	0.935
Pseudo-second-order	*k*_2_	6.84 × 10^−3^ min^−1^
	*q*_e_	27.25 mg g^−1^
	*R* ^2^	0.999
Elovich model	*α*	179.33 mg g^−1^ min^−1^
	*β*	0.25 g mg^−1^
	*R* ^2^	0.956

In this study pseudo-second order model was chosen as the best fit, because it presented the highest determination coefficient [*R*^2^] of 0.999. This value is higher respect to pseudo-first order kinetic (0.935) and to the Elovich model (0.956). Therefore, the rate-limiting step of the whole process about the removal of MB by cellulose citrate from water follows a pseudo-second order kinetic adsorption with a linear relationship between *t*/*q*_*t*_ and *t*. This is a confirmation that the rate-limiting step is the chemical adsorption, in which functional groups play a central role. Under these experimental conditions the adsorption rate is not dependent from the concentration of the adsorbate but only from the adsorption capacity.^68^

### 3.3 Adsorption thermodynamics

**Fig. 4:**
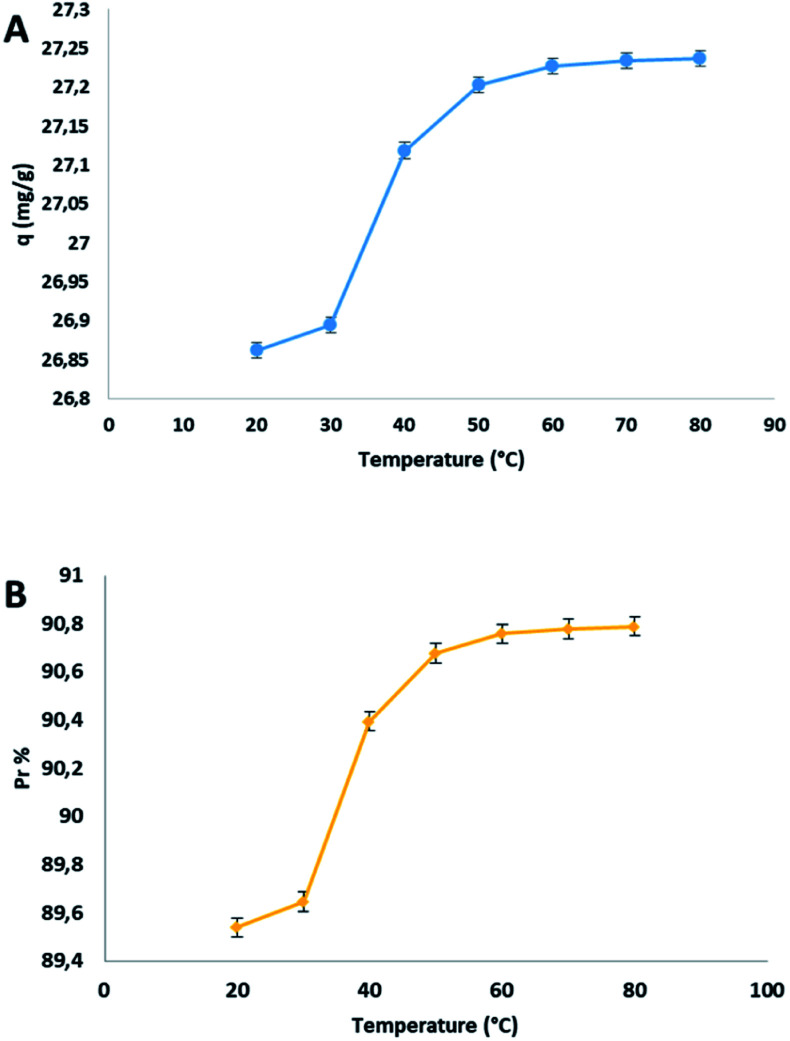
(A) Effect of temperature on the adsorption capacity *q* and (B) on the percentage removal *P*_r_.

The dependency between adsorption and temperature was evaluated by the following parameters: heat of adsorption, free energy and entropy. The effect of temperature on the adsorption capacity (*q*) and on the percentage of adsorption *P*_r_% was studied from 20 to 80 °C and the trend was reported in the following plot Fig. 4.

The adsorption capacity *q* respect to the increasing temperature, enhanced from 26.86 to 27.24 mg g^−1^, in the range of temperature indicated. This behaviour was justified by the endothermic nature of the process together to the partial opening of the pores of the cellulosic materials subjected to a temperature rise. The adsorption capacity increased significantly from 20 to 50 °C, and after that, there is an equilibrium surely due to the saturation of the adsorbent. To determinate the spontaneity of the process, the thermodynamic parameters were calculated from the van't Hoff equation (see ESI file†). The values of Δ*H*° and Δ*S*° were calculated from the slope and the intercept of the graph between ln *K*_c_*versus* 1/*T*, and are 0.98 kJ mol^−1^ and 11.14 J mol^−1^ K^−1^, respectively. The values of Δ*G*° at different temperatures were reported in Table 3.

**Table 3:** Thermodynamics parameters for methylene blue adsorption on cellulose citrate^a^

Δ*G*°	−2.3	−2.4	−2.5	−2.6	−2.7	−2.8	−2.9
*T*	293	303	313	323	333	343	353

a Δ*G*° is expressed in kJ mol^−1^ and temperature in kelvin.

The positive value of Δ*S*° is the direct consequence of the different rearrangement of the adsorbent and adsorbed after the process, having different energetic profiles. The positive value of Δ*H*° is due to the endothermic nature of the adsorption mechanism. The negative values of Δ*G*° indicate that the process is spontaneous at all used temperature. How reported in a previous study,^69^ in the case of very diluted solution in which the concentration of the adsorbate is low, it is reasonable to calculate Δ*G*° and subsequently Δ*H*° and Δ*S*° using *K*_c_ as the thermodynamic equilibrium constant. For a wider investigation, we reported another more general methodology for the calculation of the thermodynamic parameters, in which the thermodynamics equilibrium constant is approximated to the Langmuir equilibrium constant.^70,71^ In this case the value of *K*_L_ expressed previously in L mg^−1^ was multiplied by 1000 to convert the units in L g^−1^ and then multiplied by the adsorbate's molecular weight to transform *K* in L mol^−1^. Then, considering the activity coefficient of the adsorbate 1 and regarding that, the unitary activity of pure adsorbate is 1 mol L^−1^ by definition, the equilibrium constant becomes dimensionless.^72^ The values of *K*_L_ were used to calculate Δ*H*° and Δ*S*° from the slope and the intercept of the graph between ln *K*_L_*versus* 1/*T*. The values of Δ*G*° at three different temperatures, with this method of calculation, were reported in Table 4.

**Table 4:** Thermodynamics parameters using the Langmuir equilibrium constant

*T* (K)	293	303	313
Ln *K*_L_	4.87	4.92	4.98
Δ*G*° (kJ mol^−1^)	−11.9	−12.4	−12.9
Δ*H*° (kJ mol^−1^)		4.2	
Δ*S*° (kJ mol^−1^ K^−1^)		0.055	

The value of Δ*H*° of 4.2 kJ mol^−1^ was higher respect to the previous method demonstrating the endothermic nature of the process. The higher value of Δ*S*° of 0.055 kJ mol^−1^ K^−1^ remarked the strong rearrangement due to the interaction between adsorbent and adsorbate at the interface of the solution. The more negative values of Δ*G*° emphasized even more the spontaneity of the process.

### 3.4 Adsorption isotherms

**Fig. 5:**
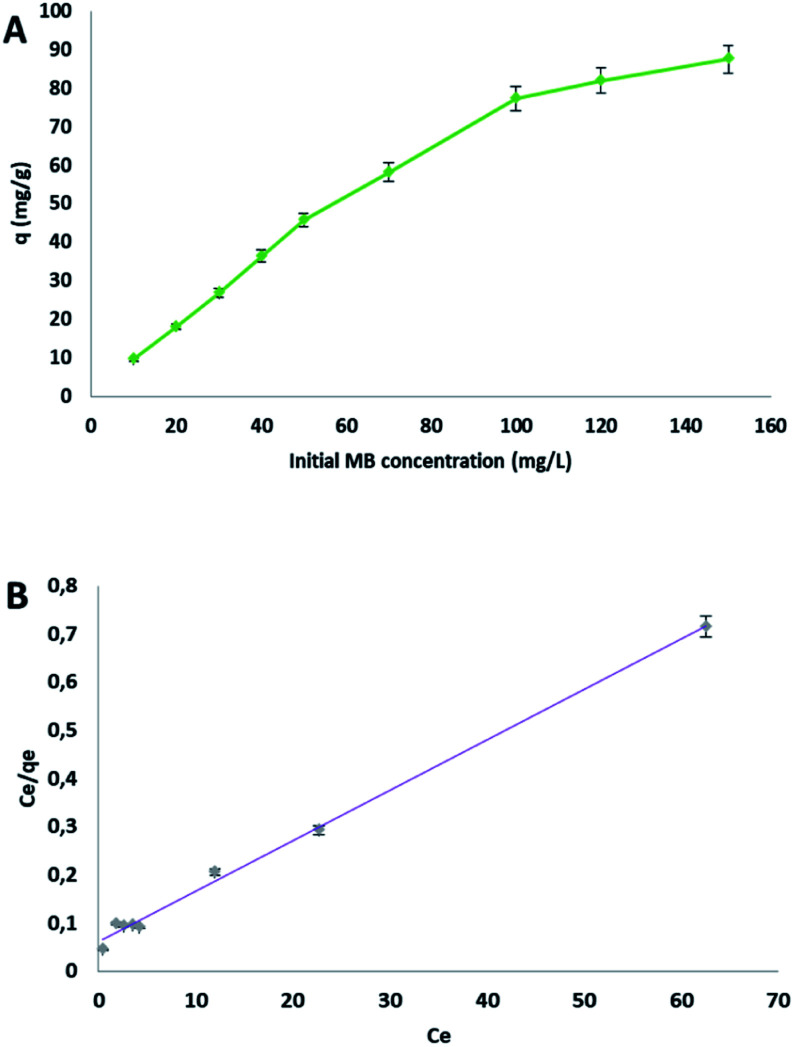
(A) Effect of the initial MB concentration on the adsorption capacity *q*, (B) Langmuir plot (*C*_e_ and *q*_e_, MB concentration and adsorption capacity at equilibrium, respectively).

The effect of the initial concentration on MB adsorption by cellulose citrate, was evaluated from 10 to 150 mg L^−1^ (Fig. 5).

At the lowest concentration of 10 mg L^−1^ the removal of methylene blue was almost complete; this result surely depends on the wide excess of the adsorbent sites respect to the dye molecules. The adsorption capacity *q* increased significantly from 10 to 100 mg L^−1^, after that there was a characteristic plateau on the plot, because of the achievement of the equilibrium (Fig. 5(A)). Three isotherms were evaluated to fit the obtained data: Langmuir (Fig. 5(B)), Freundlich and Temkin. The parameters obtained are reported in Table 4. The best determination coefficient [*R*^2^] was obtained from the Langmuir model, with a value of 0.996. The dimensionless constant *R*_L_, which is an equilibrium parameter that defines if the process is favourable or not, in all cases lied between 0.03 and 0.31 (see Table S1 in ESI file† for calculated values at all concentrations), confirming a favourable Langmuir isotherm. The test conducted starting from a MB solution of 10 mg L^−1^ furnished an adsorption capacity *q* of 9.57 mg g^−1^. From the Langmuir model, the maximum adsorption capacity obtained was 96.2 mg g^−1^. There are different materials reported in literature with higher values of the adsorption capacity, but they are more complex or made from expensive reagents.^73–75^ The result indicates that the kinetic of the process follows a monolayer adsorption due to a finite number of identical sites (carboxylic groups), which are distributed in a homogeneous manner on the surface of the adsorbent. For this reason, the other models (see ESI file†) did not produce a good fit (Table 5).

**Table 5:** Isotherm parameters for methylene blue adsorption on cellulose citrate (MB-CC)

Isotherm	Parameter	Value
Langmuir	*K*_L_ (L mg^−1^)	2.2 × 10^−1^
	*q*_m_ (mg g^−1^)	96.2
	*R* ^2^	0.996
Freundlich	*K*_F_ (mg g^−1^) (L mg^−1^)^1/*n*^	19.38
	*n*	2.44
	*R* ^2^	0.872
Temkin	*K*_T_ (L mg^−1^)	1.73
	*β*	19.62
	*b*	124.35
	*R* ^2^	0.968

### 3.5 Effect of pH

**Fig. 6:**
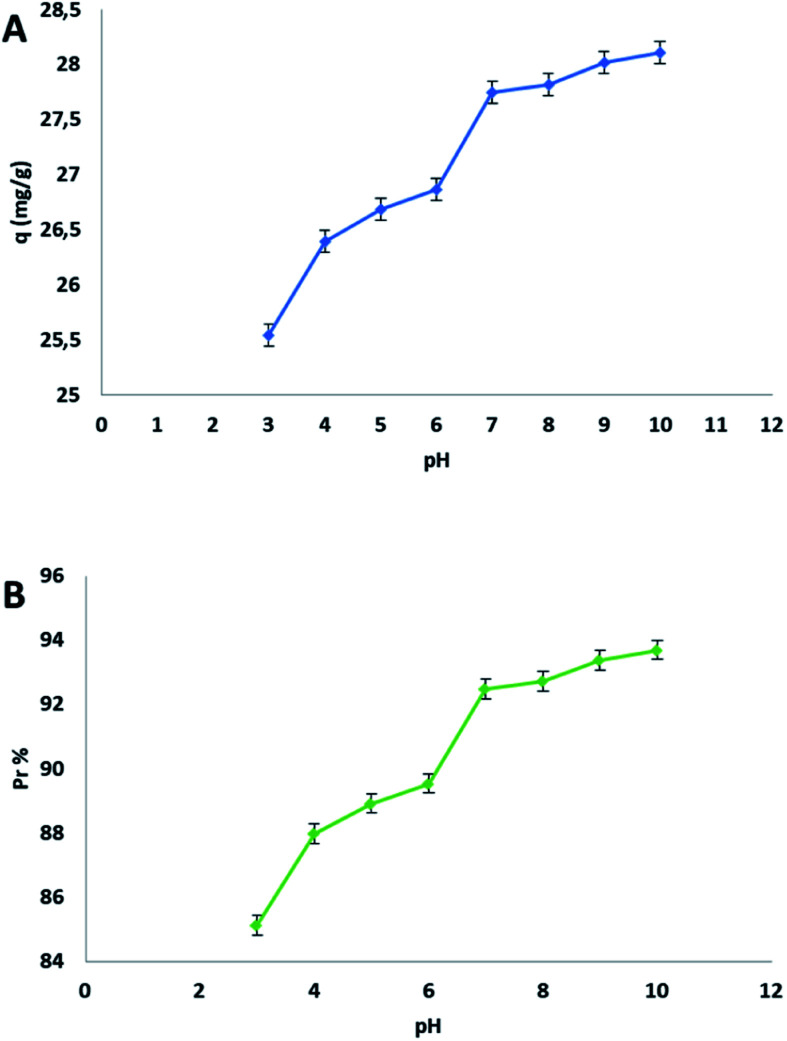
(A) Effect of the initial pH on the adsorption capacity *q*, (B) on the percentage removal *P*_r_.

The presence of the carboxylic groups in the structure of the adsorbent makes the process dependent from the pH of the starting solution. This dependency is due to the ionization equilibria that are related to the charge of the surface. The effect of the initial pH on the adsorption capacity *q* and on the percent of adsorption *P*_r_% was studied using an aqueous MB solution of 30 mg L^−1^, in a pH interval from 3 to 10. In this process the pH was a very important parameter how evidenced in the following plot (Fig. 6).

The study of pH proved that cellulose citrate is a good candidate for the adsorption of methylene blue from water in all three ranges of pH (acidic, neutral, basic). The value of *q* is already remarkable at pH of 3, because there is an ion-exchange between proton and MB cation for each carboxylic group, even if the citrate groups (carboxylic groups) are protonated at this pH value. The evidence of this mechanism is the change in the pH of the final solution after MB adsorption, which is more acidic with respect to the initial value. The value of *q* is lower at this pH value because the excess of protons in the solution produces competition with methylene blue cations. There is no evidence of pH change when the pH of the starting MB solution is above 6, because from this point, the citrate groups of the adsorbent are totally ionized in the deprotonated form. As a consequence, no ion-exchange and pH variation occur during the process, but there are only electrostatic interactions between the citrate ions and methylene blue cations for the adsorption. On the basis of the previously mentioned reasons, the best adsorption capacity *q* was reached in the basic range of the plot (Fig. 6(A)). It is remarkable to highlight that, this bio-adsorbent present an excellent adsorption capacity in a range of pH from 3 to 10.

### 3.6 Regeneration and reuse

**Fig. 7:**
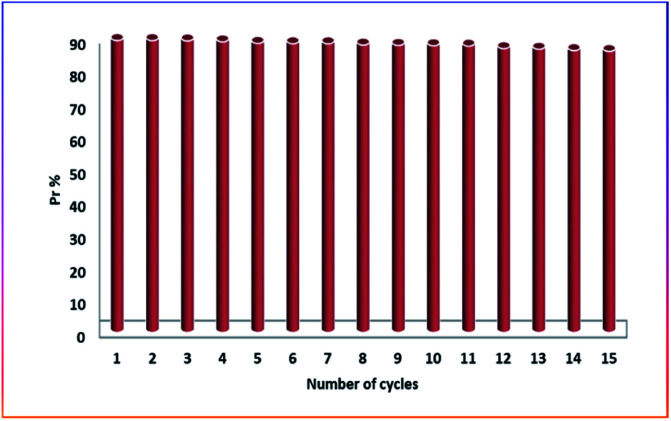
Number of regeneration cycles respect to the percentage removal *P*_r_.

The adsorbent was regenerated in this study using a dilute HCl solution (0.1 M). The desorption was instantaneous after mixing the powder of methylene blue adsorbed on cellulose citrate and the dilute acid solution. The solution was filtered through a sintered glass filter and the micro-particles of the adsorbent washed several times with distilled water before collected and dried in an oven at 80 °C for overnight time. The retained adsorption capacity was proved for 15 consecutive cycles (Fig. 7). The excess of protons of aqueous HCl solution allowed a back-exchange with MB cations to restore the protons of the citrate groups. The mild acidic solution does not affect the citrate groups and nor the cellulose chains of the adsorbent, because these chemical decompositions require generally more severe conditions. After 15 cycles the adsorption capacity was 25.86 mg g^−1^ that corresponds to a *P*_r_ of 86%.

### 3.7 Zeta potential and schematic representation of the adsorption mechanism of MB by cellulose citrate

**Fig. 8:**
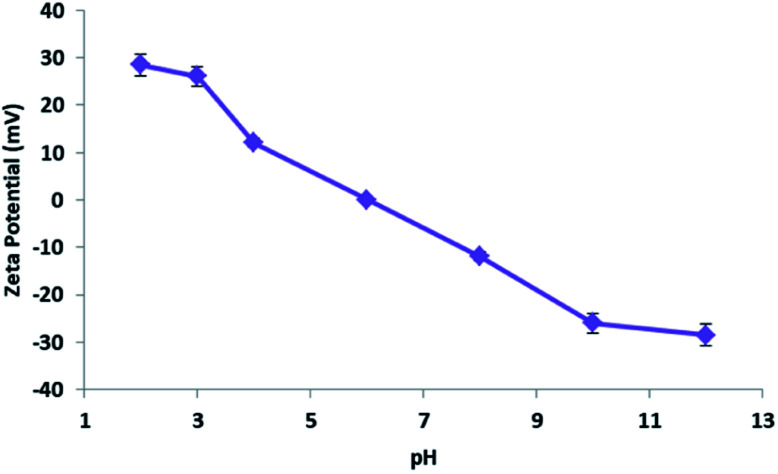
Graph of the zeta potential of cellulose citrate respect to the pH.

At this point, based on the well-known dissociation constants of citric acid and its relative ionization equilibria in aqueous solution,^76,77^ we hypothesized a mechanism for methylene blue removal by cellulose citrate supported by the calculation of the zero potential. The zeta potential furnished the surface charge density of the adsorbent. In Fig. 8 we reported the graph that shows the variation of the zeta potential of cellulose citrate respect to the pH.

**Scheme 1:**
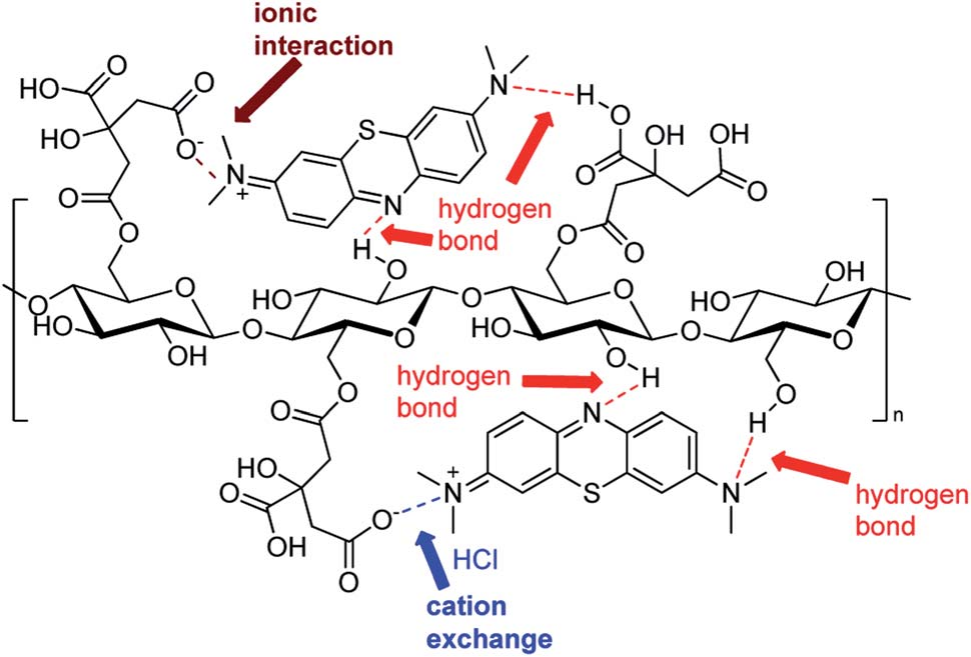
Schematic representation of MB adsorption on cellulose-citrate depending on multiple interactions.

Due to the many carboxylic groups present on the surface of the adsorbent together to the free OH groups of the glucose units, the value of zeta potential becomes negative with higher values of pH. The maximum zeta potential is −28.5 that is comparable to other similar adsorbents.^78–81^ The zeta potential at neutral pH is −3.8 that is consistent with the affinity of the adsorbent for cationic molecules like MB. At high pH (higher than pH_ZPC_) the absorption is mainly driven by electrostatic interactions between opposite charges. At low pH (lower than pH_ZPC_) the surface of the adsorbent is positively charged and the attractions are mostly due to hydrogen bonds between the adsorbent and methylene blue. In addition, together to electrostatic interactions and hydrogen bond, we hypothesized that the process of methylene blue adsorption on cellulose citrate it is a combination of multiple interactions. Depending from the pH, cellulose citrate can be protonated, deprotonated or in an intermediate form. In addition, the core of the adsorbent is both hydrophilic and hydrophobic, that is one of the main properties of cellulosic adsorbent. For this reason, we assume that the adsorption of MB on cellulose citrate takes place through different forces, including: electrostatic interactions, hydrogen bond, proton exchange, and hydrophobic interactions.^82–85^ We reported a schematic depiction of methylene blue adsorbed on cellulose-citrate (Scheme 1).

## 4 Conclusions

In this work, we proved the great efficacy of cellulose citrate as a convenient and reusable bio-adsorbent for the removal of methylene blue (MB) from artificially contaminated water. Methylene blue is used as a model in this study. The removal capacity of this bio-adsorbent for this cationic dye is a promising starting point to extend this application to other cationic pollutants present in the environment. For this reason, there is a direct correlation to the real world impact. Cellulose citrate is a biocompatible material produced by an easy, fast and cheap procedure that makes use of cellulose and citric acid as precursors. These reagents are widely available in nature and are fully biodegradable. By the studies carried out, it is possible to remark that the removal of the MB is almost complete at low concentrations and remains significant also at higher concentrations, quite independently from pH solution. Moreover, the bio-adsorbent can be regenerated many times keeping almost the same capacity, giving rise to a highly eco-compatible procedure for water remediation. Therefore, we can conclude that cellulose citrate can be considered as an optimal and green candidate for the removal of cationic dyes and pollutants in general from the environment.

## Author contributions

Fabrizio Olivito: conceptualization, writing – original draft, methodology, formal analysis, data curation. Vincenzo Algieri: investigation, methodology, data curation; Antonio Jiritano: methodology, formal analysis, data curation; Matteo Antonio Tallarida: methodology, data curation; Antonio Tursi: formal analysis, data curation; Paola Costanzo: investigation, writing – review & editing; Loredana Maiuolo: conceptualization, investigation, resources, writing – original draft, writing – review & editing; Antonio De Nino: conceptualization, investigation, resources, writing – original draft, writing – review & editing; funding acquisition.

## Conflicts of interest

There are no conflicts to declare.

## Supplementary Material

RA-011-D1RA05464C-s001
