# Supplementary material for: Cellulose citrate: a convenient and reusable bio-adsorbent for effective removal of methylene blue dye from artificially contaminated water
Source: RSC Adv. 2021 Oct 22;11(54):34309–18. doi: 10.1039/d1ra05464c (PMC9042361; doi:10.1039/d1ra05464c)
Supplement: RA-011-D1RA05464C-s001 [file RA-011-D1RA05464C-s001.pdf]

Electronic Supplementary Material (ESI) for RSC Advances.
This journal is © The Royal Society of Chemistry 2021

Electronic Supplementary Information (ESI)

Cellulose Citrate: a Convenient and Reusable Bio-adsorbent for Effective Removal of Methylene Blue Dye from Artificially Contaminated Water

*Fabrizio Olivito,[*1] Vincenzo Algieri,[*1] Antonio Jiritano,[1] Matteo Antonio Tallarida,[1] Antonio Tursi,[2] Paola Costanzo,[1] Loredana Maiuolo[1] and Antonio De Nino[*1]*

Corresponding authors email addresses

fabrizio.olivito@unical.it, vincenzo.algieri@unical.it, denino@unical.it

Adsorption kinetics………2

Adsorption isotherms ………3

Equations……4

Adsorption kinetics

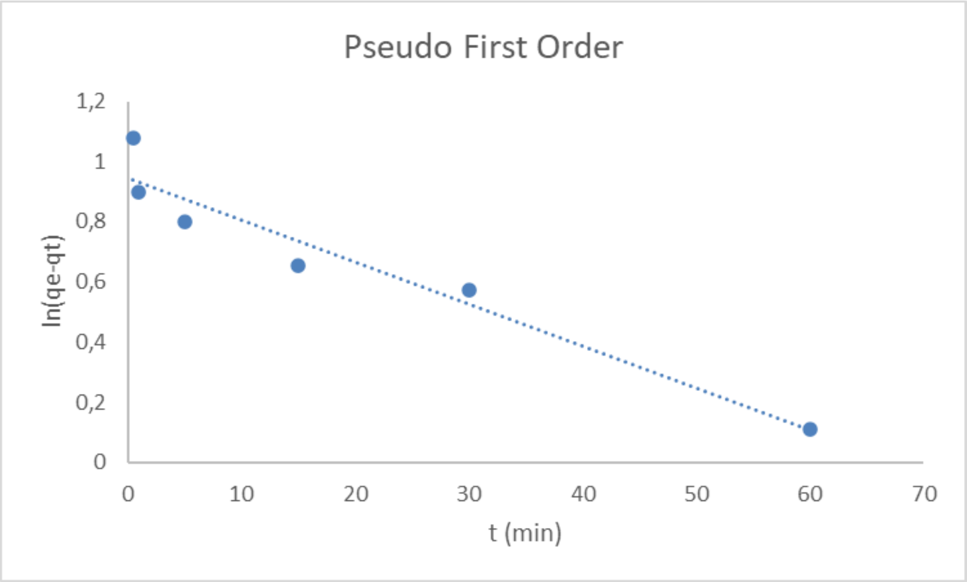

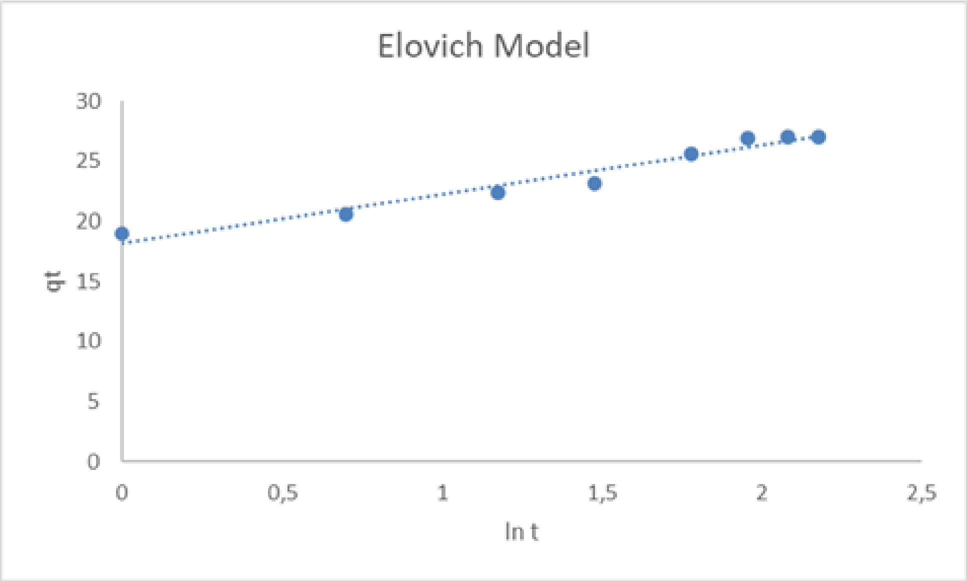

Fig. S1 Pseudo first order and Elovich model plots

Adsorption isotherms

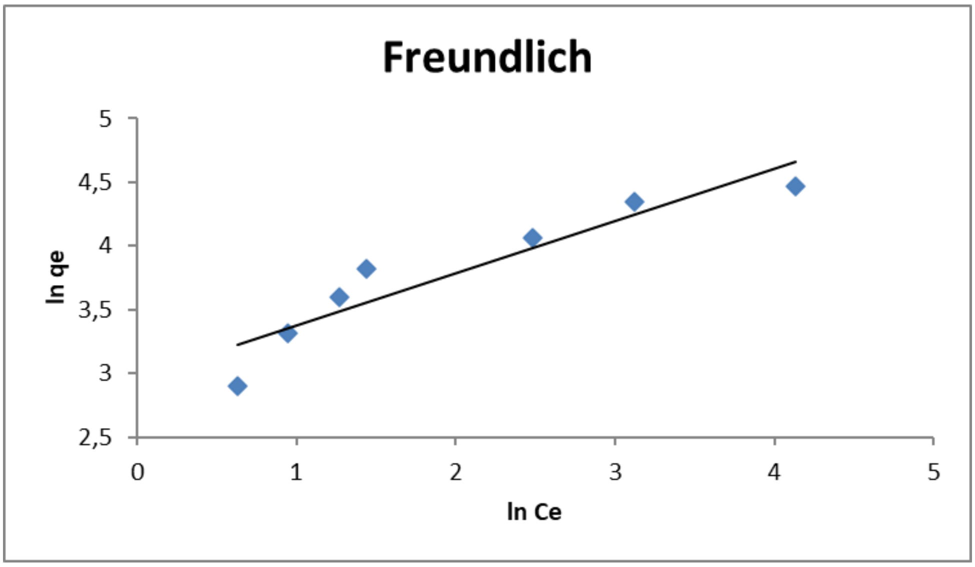

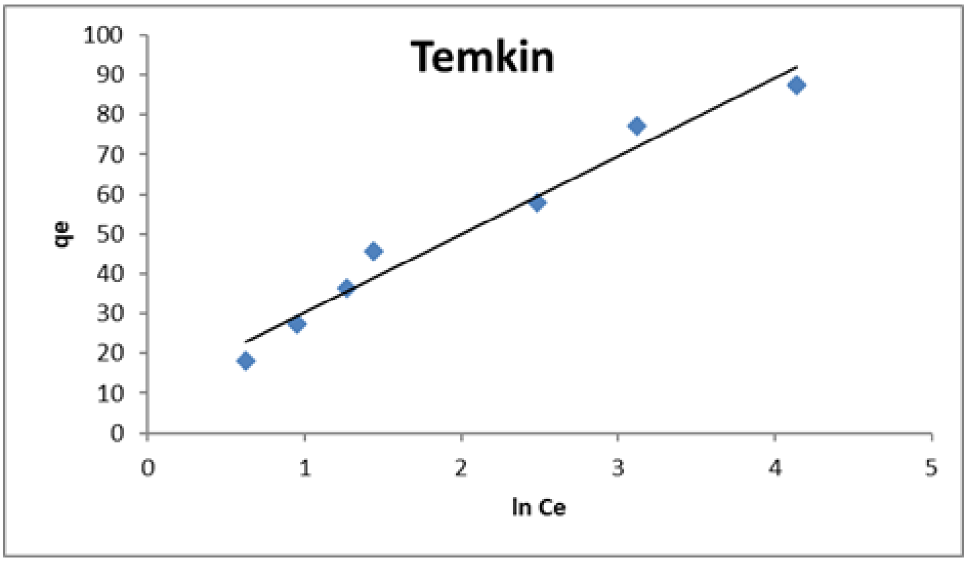

Fig. S2 Freundlich and Temkin isotherms plots

Equations

Different equations used in the study:

$$\text{Pseudo} - \text{first order equation: } ln(q_e - q_t) = lnq_e - k_1 t \quad (1)$$

$$\text{Pseudo} - \text{second order equation: } {}^{t}/_{q_t} = {}^{1}/_{k_2 q_e^2} + ({}^{1}/_{q_e})t \quad (2)$$

$$\text{Elovich equation: } q_t = \frac{1}{\beta} ln(\alpha\beta) + \frac{1}{\beta} lnt \quad (3)$$

Where, q_e and q_t are the amounts of dye adsorbed on cellulose-citrate (mg/g) at equilibrium and at time t. k_1 (min^{-1}) and k_2 (g/mg/min) are the pseudo-first order rate constant and the pseudo-second-order rate constant. α ($mg\ g^{-1}\ min^{-1}$) is the initial adsorption rate and β ($g\ mg^{-1}$) is the relationship between the degree of surface coverage and the activation energy involved in the chemisorption.

$$\text{van't Hoff equation: } lnK_c = \frac{\Delta S^o}{R} - \frac{\Delta H^o}{RT} \quad (4)$$

where, ΔS^o, ΔH^o and R represent entropy change, enthalpy change and the universal gas constant (8.314 J/mol K) respectively. T (K) is the absolute temperature and K_c (L/g) is the standard thermodynamic equilibrium constant, which is expressed by

$$K_c = \frac{q_e}{C_e} \quad (5)$$

where, q_e is the amount of adsorbed MB dye per unit mass of adsorbent at equilibrium (mg/g) and C_e is the equilibrium aqueous concentration of MB.

Further, the value of the Gibbs free energy change **$\Delta G°$** (J/mol) is calculated as:

$$\Delta G^o = -RTlnK_c \quad (6)$$

The negative value of **$\Delta G°$** indicates the spontaneity of a chemical reaction.

$$\text{Langmuir isotherm: } \frac{C_e}{q_e} = \frac{1}{k_L q_m} + \frac{1}{q_m} C_e \quad (7)$$

$$\text{Freundlich isotherm: } lnq_e = lnk_F + \frac{1}{n} lnC_e \quad (8)$$

$$\text{Tempkin isotherm: } q_e = \beta lnk_T + \beta lnC_e \quad [\text{where, } \beta = RT/b] \quad (9)$$

where the Langmuir constants q_m and k_L represent the maximum adsorption capacity of the adsorbent and the constant energy related to the heat of adsorption, while C_e (mg/L) is the concentration of adsorbate in the liquid phase at equilibrium and q_e (mg/g) is the amount of adsorbate adsorbed on the solid phase at equilibrium. k_F (mg/g) $(L/mg)^{1/n}$ indicates the adsorption capacity, and n reflects the intensity of adsorption according to the Freundlich theory. The constant β(L/mg) is related to the heat of adsorption, k_T (mg/L) is a constant of the Tempkin isotherm, b

(J/mol) is the energy constant of the Tempkin isotherm, R (8.314 J/K mol) is the gas constant and T (K) is the absolute temperature.

One of the essential characteristics of the Langmuir isotherm can be expressed by a dimensionless constant, separation factor, R_L, defined as follows:

$$R_L = \frac{1}{1 + k_L C_0} \qquad (12)$$

The value of R_L indicates the type of the isotherm; which is unfavourable ($R_L > 1$), linear ($R_L = 1$), favourable ($0 < R_L < 1$) or irreversible ($R_L = 0$). [1]

In Table S1 R_L values for each used concentration are reported:

Table S1. Values of R_L at different concentrations

C_0	R_L
10	0.31
20	0.18
30	0.13
40	0.10
50	0.08
70	0.06
100	0.04
120	0.04
150	0.03

References

[1] Kumari, S., Chauhan, G. S., Ahn, J.-H. *Chem. Eng.* 2016, **304**, 728.
